# Targeting MARylation and DePARylation in Cancer Therapy: New Promising Therapeutic Opportunities

**DOI:** 10.3390/cancers17244011

**Published:** 2025-12-16

**Authors:** Vanesa Cabeza-Fernández, Francisco Javier Ríos-Sola, David Martín-Oliva, Jerónimo Borrego-Pérez, Francisco Javier Oliver, José YéLamos, José Manuel Rodríguez-Vargas

**Affiliations:** 1 Instituto de Parasitología y Biomedicina López-Neyra, Consejo Superior de Investigaciones Científicas (CSIC), 18016 Granada, Spain; vanesacabeza@ipb.csic.es (V.C.-F.); joliver@ipb.csic.es (F.J.O.); 2 German Cancer Research Center, 69120 Heidelberg, Germany; franciscojavier.riossola@dkfz-heidelberg.de; 3 Department of Cell Biology, Universidad de Granada, 18071 Granada, Spain; dmoliva@ugr.es (D.M.-O.); jerobope@ugr.es (J.B.-P.); 4 Excellence Unit Modelling Nature, Universidad de Granada, 18071 Granada, Spain; 5 Immunology Unit, Department of Pathology, Hospital del Mar (IMIM), 08003 Barcelona, Spain

**Keywords:** PARylation, MARylation, DePARylation, PARG, MART, cancer treatment

## Abstract

The poly(ADP-ribose) polymerase (PARP) family constitutes a major group of proteins and enzymes essential for the maintenance of cellular homeostasis under physiological conditions and plays a pivotal role in the onset and progression of multiple pathological states. Members of the PARP family are classified into distinct subgroups based on their subcellular localization, structural organization, and ADP-ribosyltransferase activity. To date, the majority of studies have focused on DNA-dependent PARPs, owing to their well-established involvement in DNA repair mechanisms, cell cycle regulation, and diverse human pathologies. Nevertheless, over the past decade, a smaller subset of PARPs—limited in both abundance and enzymatic activity—has emerged as a critical regulator of numerous cellular processes, including embryonic development and disease progression. Within this subset, mono(ADP-ribosyl) transferases (MARTs) have gained growing attention as potential therapeutic targets in cancer, cardiovascular disorders, and neurodegenerative diseases. The ADP-ribose (ADPr) cycle, which comprises both branched poly(ADP-ribose) (PAR) polymers and mono-ADP-ribose moieties present either in free form or covalently bound to cellular substrates, is tightly regulated to ensure cellular homeostasis. This regulation relies on a finely tuned balance between ADP-ribosylation, DePARylation, and the subsequent recycling of mono-ADP-ribose. In this review, we provide a comprehensive overview of the biological roles of mono-ADP-ribosylation (MARylation) and DePARylation, with particular emphasis on their contribution to cancer-related processes. In addition, we discuss emerging evidence supporting their translational relevance and therapeutic potential. In conclusion, MARylation and DePARylation represent two increasingly recognized regulatory pathways whose expanding clinical significance highlights the need for deeper mechanistic understanding and further exploration in both basic and translational research.

## Simple Summary

ADP-ribosylation is a post-translational modification that plays a crucial role in DNA repair, transcription, replication, and overall cell fitness. The ADP-ribose cycle represents one of the most tightly regulated biochemical pathways in the cell, as precise control of mono- and poly-ADP-ribose levels, together with the coordinated activity of the enzymes that synthesize and recycle ADP-ribose to NAD^+^, is essential for cellular homeostasis. Moving beyond the traditional cancer framework focused exclusively on oncogenes and tumor suppressor genes, ADP-ribose can be viewed as a central signaling molecule that regulates proteins involved in tumor progression and adaptation to stress conditions within the tumor microenvironment. In this context, proteins whose roles in ADP-ribosylation are relatively less characterized, such as mono-ADP-ribosyltransferases (MARTs) and poly(ADP-ribose) glycohydrolases (PARGs), are emerging as important regulators of these processes. As their functions and biological implications continue to be elucidated, these enzymes should be considered promising prognostic markers and potential therapeutic targets.

## 1. Introduction

ADP-ribosylation is a crucial NAD^+^-consuming pathway and post-translational modification involved in various biological processes such as DNA damage repair, transcription, and cell cycle regulation. Human tissues express 17 ADP-ribosyltransferases (ARTs) [1,2,3], with PARP (Poly ADP-ribose Polymerases) enzymes and tankyrases playing pivotal roles in signaling events. All members present, as a structural characteristic, an ADP-ribosyltransferase catalytic domain (also called PARP signature) whose interaction with additional domains determines the subcellular localization and biochemical function [2,4,5]. Considering the ability to modify their targets through a single ADP ribose (mADPr, Mono ADP-ribose (MAR)) moiety or through the addition of branched ADPr polymers (pADPr, Poly ADP-ribose (PAR)), ARTs are subdivided into mono(ADP-ribosyl) transferases (MARTs, catalyzing MARylation) and poly(ADP-ribosyl) transferases (PARPs or PARTs, catalyzing PARylation) [3,6]. Inside the PARP subgroup, PARP1-3 are key players in recognizing DNA lesion sites and initiating DNA repair cascades by transferring ADP-ribose moieties as single units (mono-ADP-ribosyl, MAR) and subsequently elongating them into poly-ADP-ribose chains (PAR) [3,6,7]. This modification, particularly the PARylation of histone tails, facilitates nucleosome remodeling, chromatin structure, and genome stability. MARTs proteins drive essential roles in the regulation of RNA metabolism, cellular transport, focal adhesion, and stress responses [8,9]. These insights have increased our understanding of the biological functions of MARTs in human diseases such as cancer, neurodegenerative, and metabolic diseases [10]. Mono-ADP-ribosylation of a protein, or alternatively the maintenance of a MARylated state following prior PARylation, does not confer the same biological significance, as the underlying target-specific MARylation mechanisms differ substantially and lead to markedly distinct functional consequences within the cell.

The clinical relevance of PARPs lies in the development of catalytic inhibitors that compete with NAD^+^ (PARP inhibitors, PARPi), effectively blocking the enzyme’s catalytic site [4]. The mechanism of action of PARPi was originally described as a synthetic lethal interaction between PARP inhibition and BRCA1/2 deficiencies in cancer cells. These inhibitors effectively target tumors with compromised homologous recombination (HR) repair, benefiting patients with breast, ovarian, and prostate cancers [1,4,5]. However, there are problems associated with the clinical advantages of PARPi, from the resistance to PARPi monotherapy to the fact that its druggability did not allow for low-dose treatments, which sometimes worsened the patient’s clinical condition and evolution.

Therefore, there is an urgent need to clearly understand and differentiate between these two modifications by ADP-ribose in order to achieve new goals in the development of applied therapies. Furthermore, several novel inhibitors of MARTs have been developed and are nearing clinical utility [2]. In the last six years, the therapeutic value of its inhibition has not been fully understood and awaits further research into its role in cancer aggressiveness and other pathologies.

DePARylation, or the enzymatic removal of covalent modifications by poly ADP-ribose, performed by ADP-ribosylhydrolases, is considered an essential reaction not only for the recycling of ADP-ribose, thus maintaining homeostasis in NAD^+^ metabolism, but also as the on/off switch for a multitude of biochemical pathways. The main PAR erasers are the poly(ADP-ribose) glycohydrolases (PARGs), responsible for the degradation of PAR, breaking it down into ADP-ribose and releasing it from the target protein [11,12]. PARG activity remains single meiotic ADP-ribose into the targets, subsequently eliminated by the other ADP-ribosylhydrolases [2,13]. The therapeutic potential of regulating the amount of ADP-ribose at a specific moment in the cell cycle or during the activation of a metabolic pathway is enormous, implying that PARG inhibition should be considered a therapeutic target with great translational potential [1,14].

All of the above leads us to define the ADP-ribose cycle, involving three main groups of proteins: writers, readers, and erasers. Writers (ARTs) add ADP-ribose units to targets from NAD^+^; biochemically, PARylation/MARylation processes must be finely regulated, given that excessive PARylation/MARylation can affect the cell’s energy status due to the high cost of ATP and NAD^+^ required. In a pathological scenario where resource monopolization and metabolic reprogramming are so important, such as cancer, the activities of these writers of ADP must be controlled. Readers (PAR/MAR-binding proteins) containing specific domains, like macro domains, WWE domains, or PAR/MAR-binding motifs, can recognize and bind to the ADP-modified proteins. This binding can have various effects, such as activating or repressing the target protein’s function. Erasers (ADP-ribosylhydrolases), including PARG, remove the ADP-ribose units from targets, effectively reversing the modification. This process helps regulate the levels of ADP-ribosylation and its downstream effects, forming a promising way for developing inhibitors with applications in human diseases [4,13].

In conclusion, in this review, we will clarify the main therapeutic advances related to two of the three groups of actors involved in the ADP-ribose cycle in the cell. Specifically, we will focus on the translational power of mono-ADP-ribose writers and the proteins responsible for erasing and recycling PAR modifications. This will emphasize the importance of maintaining MARylated proteins or, if necessary, maintaining them modified by a final ADP-ribose residue.

## 2. MART Enzymes Mediate Physiological and Pathological Processes in Cells

**Figure 1:**
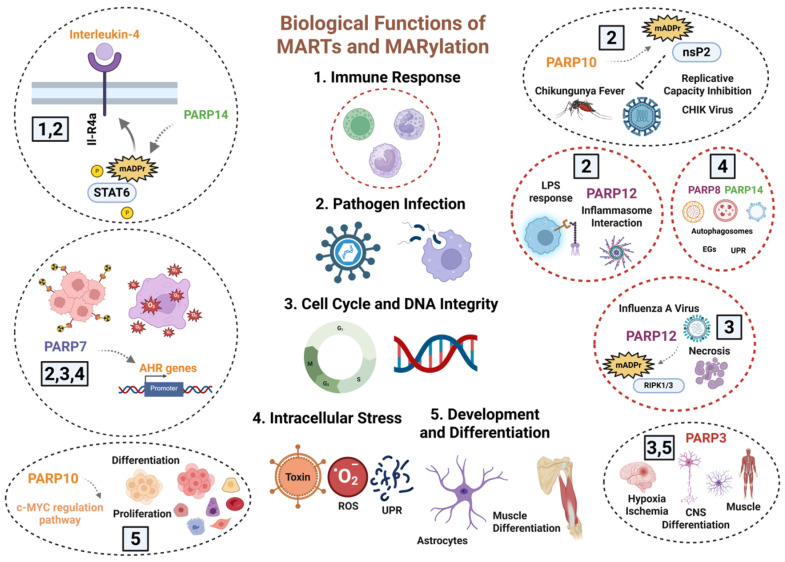
Overview of Biological Functions of MARTs and MARylation: Cytosolic MARTs modulate several biological processes, such as DNA Repair Pathways, Mitotic Progression and Cell Cycle Regulation, Virus Infection, Inflammation, and Organelle Response to Stress. A broad group of MARTs regulates the immune response, sometimes through the inflammatory cascade, and the tissue response to pathogen infections, as is the case with PARP7/10/12 and 14. Similarly, PARP7 and PARP12 modulate the correct development of the mitotic cycle; in this function, PARP3 also controls cell cycle stability and mitosis. Through their role as regulators of detoxification capacity, PARP7/8 and 14 modulate the elimination of organelles, misfolded proteins, and the response to ROS. Finally, other MARTs such as PARP3 and PARP10 are involved in tissue differentiation and development. To date, their action, through MARylation, is attributed to PARP10, PARP12, and PARP14, although there is disagreement as to whether PARP3 modifies its described targets in cell cycle and differentiation through ADP-ribose oligomers. Created in BioRender. Rodríguez Vargas, J.M. (2025) https://BioRender.com/02c6ion (accessed on 10 December 2025).

Mono(ADP-ribosyl) transferases (MARTs) constitute a subclass of the poly(ADP-ribosyl) polymerase (PARP) family [15]. Based on sequence homology with the PARP-1 active domain (PARP signature), MARTs are sub classified into 3 large groups: (i) RNA Binding CCCH Zn Finger PARPs (PARP7/PARP12/PARP13), (ii) Macro Domain Containing PARPs (PARP9/PARP14/PARP15), (iii) PARPs with no sub-classification (PARP4/PARP6/PARP8/PARP10/PARP11/PARP16) [15]. The wide variety of structural domains and subcellular locations among MART enzyme members, as well as their disparate affinity for the substrate NAD^+^ to synthesize ADP-ribose, result in a broad range of potential protein substrates and residues targeted by mADPr modification. MARTs modify their target proteins by the addition of a single ADPr moiety in a reaction called mono(ADP-ribosyl) ation (MARylation) [9,16]. Although the cellular location and function of DNA-dependent PARPs and tankyrases are well defined, with their main function being to maintain the architecture, stability, and function of the nuclear genome, despite increasing interest in mono(ADP-ribosyl) transferases (MARTs) and MARylation, their precise biological functions remain poorly understood. This limited understanding paradoxically underscores their strong therapeutic promise, positioning MARTs as attractive targets across a wide spectrum of human pathologies and opening an exciting and rapidly expanding field of investigation [2,9]. MARTs are generally characterized by being cytosolic enzymes, whose function, location, and affinity for the NAD^+^ substrate are variable depending on the member we study [8,9] (Figure 1). Cytosolic MARTs recognize a wide variety of residues in their targets (such as Ser, Asp, Glu, and Cys), which has allowed the development of tools to identify targets, physiological and pathological functions, and the development of small molecules with therapeutic capacity against these enzymes [10,11].

The major limitation to research into MARylation has been the inability to detect modifications by mono ADP-ribose (MAR) residues and distinguish them from PARylation. Historically, studies with PARP knockout cell models and in vitro radioactively- or biotin-labeled NAD^+^ allowed to visualize the target modifications by poly ADP-ribose (PAR). More recently, the effective combination of omics has made it possible not only to characterize targets but also to structurally determine amino acid-targeted residues and analyze the stoichiometry of these modifications in several targets and pathological scenarios [8,9]. In recent years, new methodologies have been designed to generate protein-linked mono(ADP-ribose), oligo(ADP-ribose), and poly(ADP-ribose) (MAR, OAR, and PAR, respectively) in vitro as an auto-modification of DNA-dependent PARP enzymes, such as PARP-1 or PARP-3. Purified recombinant PARPs are incubated individually in the presence of different concentrations of NAD^+^ and sheared DNA, resulting in various (auto-) mono- and poly-ADP-ribosylation [17,18]. Nevertheless, further studies at the level of structural biochemistry and in silico modeling are needed to determine the functions, targets, and clinical relevance of MAR-based modifications. All of these advances have highlighted the development of effective therapies. Current experimental approaches to detect MAR and residues susceptible to MARylation are based on two critical points: 1. Identifying mono-ADP-ribosylation related to the function and localization of protein, DNA, and RNA. 2. Generating protein-linked and protein-free mono-, oligo-, and poly(ADP-ribose) in vitro [8,18,19]. Considering these molecular premises, we can define two large groups of methodologies.

Labeled Molecules to detect MARylation: Because NAD^+^ is not permeable to the cell membrane, the use of biotin-labeled NAD^+^ has been limited to in vitro assays. In this way, two strategies allow reliable detection of molecules subject to modification by mADPr. The most prominent examples would be the detection of mono ADP-ribosylated proteins based in an alkyne-adenosine analog, N6-propargyl adenosine (N6pA) [20] and a recently developed method, called Enzymatic Labeling of Terminal ADP-ribose or ELTA, based to use the human enzyme 2′-5′ oligo adenylate synthetase 1 (OAS1) to for labeling free or protein-conjugated ADP-ribose monomers and polymers [21,22]. ELTA enables high-precision biophysical measurements of protein binding to ADP-ribose residues, oligomers, and even small polymers of defined lengths in DNA and protein targets. It also allows specific lengths of pADPr and mADPr to be associated with targets at specific phases of the cell cycle or at well-defined stages of cell development or proliferation [23,24,25,26].

New Generation Antibodies to detect MARylation: The absence of MAR antibodies has been compensated through the knowledge of a multitude of domains susceptible to being modified by ADP ribose (macro domains D1/D2, WWE domains, PAR-binding zinc fingers, WGR domains, or virus structural domains) [27,28]. Recent developments in enzymatic approaches to obtain ADP-modified peptides by PARP6, PARP3, PARP14, and PARP10 in human and murine diseases are advancing our ability to generate site-specific MAR antibodies, with future application in animal models of cancer and neurodegenerative diseases [29,30,31,32].

### Biological Implications of Cytosolic MARylation

In the last three decades, the majority of projects related to the therapeutic potential of the PARP family focused on the wide range of documented functions of DNA-dependent PARPs, the main producers and transferases of ADP-ribose oligomers and polymers, as well as the significant clinical potential of their inhibition. However, in the last 10 years, numerous studies have demonstrated new and important functions of other PARPs, lacking PARP activity itself or even any documented activity as mono-ADP transferases. Thus, it has been shown that MARTs, regardless of their activity on ADP-ribose levels, are involved in processes such as RNA metabolism, cellular transport, energy metabolism, focal adhesion, and immune response (Figure 1) [4,9,33]. The main obstacle encountered when characterizing the structure, location, and functions of MARTs was the inability to detect mADPr bound to substrates, as well as to distinguish it from pADPr levels or the remnant activity of ADP-ribose eraser, which kept various biomolecules mono ADP-ribosylated [4,8,20].

Different approaches and discoveries have allowed the description of many functions associated with MARTs. In some cases, targets susceptible to being MARylated have been perfectly described; in other cases, speculation and the lack of scientific evidence have led to the identification of the consequences but not the intrinsic molecular mechanism.

Functional role of MARTs on transcription regulation: Structural studies of various proteins maintaining the architecture of DNA, such as histones and topoisomerases, provided the first indications that certain MART proteins might be actively regulating transcription processes in very specific scenarios. For example, PARP14 modifies, by mADPr, several members of the STAT (signal transducer and higher activator of transcription) factors. Specifically, it has been described that the interleukin-4 (IL4)-mediated cellular response requires PARP14 activity on STAT6 to bind to the target promoters of the IL4-mediated inflammatory cascade in cancer, inflammation, and immune response [34]. In other ways, PARP7 acts as a repressor of AHR (aryl hydrocarbon receptor)-mediated transcription. ARH is a ligand-activated transcription factor that regulates gene expression in response to environmental contaminants, playing a key role in detoxification and immunity in response to dioxins, unfolded proteins, and virus infection [20,35]. PARP10 regulates the MYC-induced transcription, influencing genes involved in cell cycle, proliferation, and differentiation, consequently playing a key role in several cancers [20,36]. PARP3 is a DNA-dependent PARP, whose functions have been described in the last 10 years. There is controversy as to whether it is truly a MART protein or whether, on the contrary, it is capable of synthesizing ADP-ribose oligomers. Its nuclear location is attributed to its functions in cell cycle control, telomere stability, chromosomal rearrangements, and mitotic segregation through its interaction with histones, tankyrase 1, TERF1, or PARP1. Transcriptionally, PARP3 mediates the regulation of NOX4-induced ROS and mTORC2 activation during astrocytes differentiation in response to oxidative stress and hypoxia ischemia in vivo [37] and recent findings unraveling PARP3 as a driver of efficient murine skeletal myogenesis and muscle function in young adults through the regulation of the recruitment of the repressive histone flag H3K27me3 onto a subset of muscle-differentiation related genes (Figure 1) [38].

Active Role in Immunity and Inflammation: In relation to the inflammatory and immune response during infection by various types of pathogens, mainly viruses, it has been described that certain MARTs should be considered as active modulators of both the immune response underlying the infection and the cell death processes that occur at the tissue level. PARP12 is considered a potential regulator of the immune response through its localization in stress granules formed in response to IFNγ stimulation/inflammasome, which could be related to the antiviral response [39]. In parallel, in an LPS treatment model, PARP12 has been shown to colocalize with members of the proteins that regulate autophagy and mitophagy, such as p62/SQSTM1, significantly influencing the activation of the NF-κB pathway at the transcriptional level [39]. Recent research has demonstrated that PARP12 MARylates RIPK1 and RIPK3, key kinases involved in regulating cell death pathways, favoring necrosis and inflammatory response in models of influenza A virus infection [40]. Both models highlight PARP12 as a potent modulator of the immune response to viruses, which establishes a therapeutic future based on its effective inhibition. PARP10 is generally considered a mono-ADP ribose transferase that primarily regulates cell cycle control, metabolism, and cell death processes in response to mitochondrial stress and, in most cases, during carcinogenesis [41]. Recently, several studies have demonstrated its involvement in immunity (Figure 1). A recent study has established that PARP10 MARylates the nsP2 subunit of the non-structural polyprotein nsP1-4, essential in the replication of the Chikungunya virus (CHIKV Fever), blocking its proteolytic activity and consequently altering the replicative capacity of the infectious viruses [42]. Other studies have shown that PARP10 is a repressor of nuclear factor-κB (NF-κB) signaling in models of bacterial toxin infections, through the MARylation of NEMO, which favors increased I-κB stability and less p65 translocation into the nucleus. Consequently, NF-κB is inhibited and degraded in the proteasome, blocking its transcriptional function (Figure 1) [43].

PARP4, also known as vault poly(ADP-ribose) polymerase (vPARP), is an essential component of the vault ribonucleoprotein complex. Through interactions involving its BRCT domain, PARP4 binds to vault RNA (vtRNA), thereby contributing to the selective recruitment and transport of associated proteins and molecular cargos within the vault particle; so PARP4 could modulate cytosolic-nuclear transport, as well as maintain nuclear pore stability [44]. Given the importance of vault particles in RNA transport, PARP4 must be considered a regulator of the immune response during embryonic development, detoxification of toxins in adult brains, being shared with PARP6, and resistance of tumor cells to different drugs (Figure 1) [44].

Cell Stress Response and Cellular Fitness: In recent years, MARTs and the mono-ADP-ribosylation process have emerged as important modulators of various processes related to the stability and function of organelles and, therefore, in the adaptive response of both non-transformed and tumor cells to stress situations. The connection lies in the interaction of several MARTs with non-membrane-bound cytosolic stress granules (SGs), formed in cells during stress conditions (e.g., oxidative stress, heat shock, UPR stress, organelle dysfunction) [45]. PARP8/10/12/ or 14 interacts with the surface of SGs, being able to modify by mADPr various components such as RNA-binding proteins like Ago2 and TIA1, as well as chaperones like Hsp70 and helicases such as DDX3 [20,46,47]. PARP8 and PARP10 are associated with classic oxidative stress processes due to mitochondrial dysfunction, DNA damage, and even glucose and amino acid deprivation. In this regard, there is a marked interplay with PARP1-type poly ADP-ribose transferases, due to the essential role of ADP-ribose polymers in cellular homeostasis [48]. Glycogen and glucose deprivation models have demonstrated significant involvement of PARP10, PARP8, and PARP12 in the formation of self-regulating glycogens (SGs) associated with autophagosomes (Figure 1) [48]. Amino acids and glucose deprivation impacts mono-ADP-ribosylation through its effect on NAD^+^ levels. PARP10, PARP16, and certain sirtuins (SIRT4, SIRT6, SIRT7), which possess mono-ADP-ribosyltransferase activity, compensate for the lack of PARP1 activity, essential in the initial steps of starvation-induced autophagy and preventing a total energy collapse that leads to necrosis and tissue damage. In vitro and in vivo models in Drosophila have demonstrated these observations [8,48,49]. Hypoxia, a common event during embryonic development, tissue maintenance, and tumor progression, leads to high rates of ROS production, alterations in proton flow through mitochondrial complex I, and, consequently, an imbalance in NAD^+^/NADH levels. In this sense, stress granules are common in polarized regions of the cytoplasm where there is an accumulation of mitochondria and fine communication with the Golgi and the ER [50]. It has been postulated that both mono(ADP-ribose) transferases and mono(ADP-ribosyl) ating sirtuins could modulate mitochondrial dynamics and function during periods of decreased oxidative phosphorylation. However, there are two challenges in demonstrating this involvement. In mitochondrial diseases, oxygen deprivation compensates for neurodegeneration despite mitochondrial dysfunction, both in cell and animal models. In cancer, tumor cells compensate for the loss of oxidative phosphorylation during hypoxia by inducing anaerobic glycolysis to produce ATP (Warburg effect). Both scenarios raise serious doubts about whether MARTs could balance the absence of PARP1, PARP2, and PARP3 activity in cases of PARPi treatment. This field needs to be explored in greater depth, given the undeniable relevance of MART on Golgi, ER, or lysosomes, three of the major targets during hypoxia, and with fine interconnection with this type of pathology (Figure 1).

## 3. Biological Implications of MARTs and MARylation in Tumor Progression

### 3.1. MARTs, Descriptive Tools with Great Potential in Cancer

**Figure 2:**
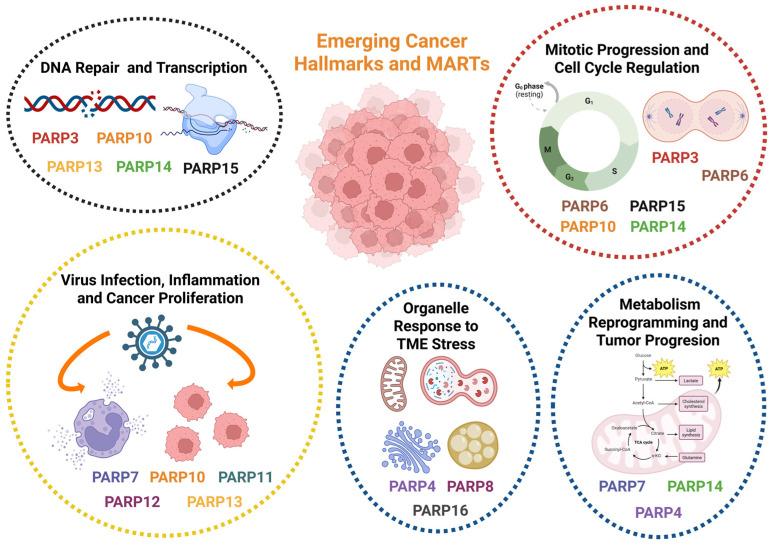
Role of MARTs in Cancer Progression: In cancer, MARTs are considered emerging therapeutic targets, finely influencing the outcome of different aspects related to tumor progression, such as transcription control, regulation of the inflammatory cascade, or metabolic reprogramming in response to TME stress (starvation, hypoxia, organelle stress, and unfolded protein accumulation). Several MARTs are considered oncoproteins whose expression is exacerbated in certain types of tumors and are therefore considered prognostic markers, such as PARP3, PARP7, and PARP8. However, others are attributed with a function as tumor suppressor proteins, as is the case with PARP4, whose mutation favors malignancy and metastasis. Overview of the main described implications of MARTs in tumor progression in relation to their TME. Created in BioRender. Rodríguez Vargas, J.M. (2025) https://BioRender.com/hiu0mjb (accessed on 10 December 2025).

Mono(ADP-ribose) transferases regulate a wide range of cellular processes, and their activity is associated with different types of tumors [8,9,51]. Recent advances have significantly expanded our understanding of the biology and function of most non-DNA-dependent PARPs. In this regard, the range of functions attributable to MARTs has increased, highlighting various essential roles in cellular fitness for each single MART, as well as compensatory events between different members. While physiological functions are of great importance, from a clinical perspective, understanding and characterizing the therapeutic implications of MARTs will allow for the identification of molecular targets and further the effective development of drugs. While most MARTs are linked to tumor promotion, some play a role in suppressing tumor development. In this new section, we will summarize the main implications of MARTs and MARylation in cancer, identifying the descriptive models and molecular tools that currently have effectiveness and clinical relevance in cancer (Figure 2). The limited availability of potent and selective MART inhibitors has positioned in silico modeling, omics-based approaches, and the generation of knockout (KO) and transient experimental models as the primary strategies for elucidating the roles of MARTs in carcinogenesis and tumor progression.

Through the development of KO and silenced transients for PARP4, PARP6, PARP12, and PARP3, functions in proliferation, drug resistance, and metastasis progression of different solid tumors have been characterized. For instance, PARP12 depletion promotes cell invasion and migration in cell models of hepatocellular carcinoma (HCC), as well as metastasis in vivo [52]. Similarly, PARP4 KO models show increased tumorigenicity in lung cancer cells [53] and higher proliferation in the HCC1143 cell line of breast ductal carcinoma [54], indicating a tumor-suppressive role for these MARTs. In other cases, such as PARP6 and PARP10, there is controversy as to whether we are dealing with a role in tumor repression and progression.

Despite their suppressive role, PARP4 expression is also associated with resistance to cisplatin treatment, as evidenced by the restoration of sensitivity in resistant ovarian cancer cells following PARP4 silencing with small interfering RNA (siRNA) [54,55].

PARP3: Among MARTs that promote tumor development, PARP3 is one of the best characterized. Beck et al. described in 2018 [56] that PARP3 silencing reduces the viability of BRCA1-deficient cells of triple-negative breast cancer (TNBC) due to increased genomic instability. Furthermore, complete knockout of PARP3 using CRISPR/Cas9 technology reduced cell proliferation, survival, and migration of TNBC cells, abolishing their tumorigenic capacity in vivo through attenuation of oncogenic signaling mediated by Rictor/mTORC2 in BRCA1-deficient TNBC cells [56,57]. Pharmacological inhibition of PARP3 with the specific inhibitor ME0328 [56,57] had a similar effect, reducing viability and mTORC2/Rictor activity in the absence of BRCA1 in TNBC cells [57]. The effect of ME0328 had previously been studied in TNBC, enabling greater sensitization of these tumor cells to the alkaloid vinorelbine used in the treatment of metastatic breast cancer, increasing apoptosis and cell cycle arrest, vinorelbine-induced microtubule destabilization, and greater inhibition of PARP3 [58] (Figure 2). PARP3 elimination has also been studied in glioblastoma cell models, but there was no significant effect on tumor proliferation and growth, although sensitivity to microtubule-destabilizing agents was significantly increased in glioblastoma cells [59].

PARP4: According to The Human Protein Atlas (https://www.proteinatlas.org/), PARP4 is considered a prognostic marker in pancreatic and renal cancer. It also shows high levels of protein expression in glioblastoma-type tumors, melanoma, colon, lung, and kidney cancer. In the last four years, several studies have confirmed PARP4 as a highly relevant therapeutic target in cancer, regardless of its attributed function. For example, it has been described that in melanoma, PARP4 migrates to the nucleus where it promotes proper DNA repair by the NHEJ system, modifying the Ku80 protein through MARylation. PARP4 deficiency sensitizes melanoma cell lines to treatment with ATM inhibitors (ATMi), thus attributing to it an active role as an oncoprotein in melanoma [60]. Similarly, Youn Sung et al. [55] analyzed PARP4 mRNA expression profiles in ovarian cancer cell lines, confirming that it is upregulated in cisplatin-sensitive cells compared to cisplatin-resistant cells. The mechanism of action is based on differential methylation profiles between ovarian cancer cell lines at the PARP4 promoter, leading to the conclusion that this MART should be considered a diagnostic marker of great interest (Figure 2) [55]. Other studies along the same lines consider PARP4 to be a breast cancer susceptibility gene; however, its function in this type of tumor is currently unknown [61]. In contrast, Fei Lee et al. published a study demonstrating that PARP4 deficiency or mutation promotes progression and malignancy in KRAS- and EGFR-driven lung cancer cells through a direct association with hnRNPM, a key protein controlling circRNA biogenesis, stability, and splicing fidelity [53]. Therefore, we can consider vPARP as one of the most clinically relevant markers among MARTs.

PARP6 and PARP10: This ambiguous role in tumor repression and progression is also observed in PARP6 and PARP10. Several studies have described PARP6 as a colorectal cancer suppressor protein [62,63]; however, Wang et al. demonstrated that its silencing not only fails to promote tumor development but also inhibits cell invasion and promotes apoptosis, showing a pro-tumor role for PARP6 in colorectal adenocarcinoma [29]. The oncogenic role of PARP6 has also been described in breast cancer, where pharmacological inhibition of PARP6 promotes apoptosis of cancer cells in vitro. However, PARP6 shows a duality, acting differently on the expression and stability of the Survivin protein, proliferation, and migration, in one case as an oncoprotein in gastric cancer models [64], and conversely, acting as a suppressor protein of malignancy in colorectal cancer models [62]. Regarding PARP10, silencing this enzyme with siRNA inhibits cell proliferation and induces apoptosis in the HSC3 cell line of oral squamous cell carcinoma (OSCC), while its metastatic activity is reduced by inhibiting the migration and invasion processes of this cell line (Figure 2) [65]. Inhibition of proliferation was also observed following PARP10 silencing via the CRISPR/Cas9 technique in acute myeloid leukemia cell lines [66] and HeLa cells, possibly due to increased replicative stress as described by Schleicher et al. in 2018 [36]. However, other studies showed controversy in the published results and conclusions, given that Zhao et al. claimed that CRISPR/Cas9 silencing of PARP10 in HeLa cells increased invasion, cell migration, and metastasis in vivo, while maintaining levels of proliferation, colony formation, cell cycle, and apoptosis, indicating a tumor progression suppressor role in this model (Figure 2) [67].

PARP7 and PARP8: PARP7 has also demonstrated a role in tumor cell growth [68,69]. Various inhibitors have been developed against this member with high selectivity and well-demonstrated pharmacokinetic properties, as well as greater inhibition of cell proliferation than RBN-2397 (a clinical candidate in Phase I) [70], both in cancer cell cultures and in vivo mouse models [71,72]. The effectiveness of PARP inhibition in tumor progression lies in restoring type I IFN signaling through PARP7 inhibition [71,73,74]. Additionally, PARP7 inhibition prevents the MARylation of FRA1, an AP-1 family transcription factor which is frequently overexpressed in tumors, leading to its destabilization, subsequent proteasome degradation, and increased apoptosis in NCI-H1975 non-small cell lung cancer cells [68]. Natural molecules like Epigallocatechin-3-gallate (EGCG), a polyphenol from green tea, have also proven effective in blocking tumor progression by inhibiting PARP7 and other MARTs, e.g., PARP16, blocking the unfolded protein response (UPR response) and endoplasmic reticulum stress, thereby inducing apoptosis in tumor cells (Figure 2) [74,75,76]. This makes EGCG an important therapeutic treatment in combination with ER stress-induced agents [75]. Similarly, to date, the role of PARP8 in tumor progression has not been determined. Recent studies indicate that PARP8 knockdown models in uveal melanoma cell lines decrease tumor cell proliferation and migration while promoting an immunosuppressive state of the tumor microenvironment, suggesting a pro-tumor role for PARP8 (Figure 2) [69,77].

PARP9, PARP11, PARP13, PARP14, and PARP15: The remaining PARP members with MAR activity exhibit a tumor-promoting role. For instance, PARP9 silencing inhibited cell migration in in vitro cultures of HCC1806 breast cancer cells [69,78] and led to an increase in the tumor suppressor IRF1 and a decrease in the expression of the proto-oncogene IRF2, inhibiting proliferation in diffuse large B-cell lymphoma [79]. The increase in IRF1 and inhibition of proliferation following PARP9 silencing have also been observed in prostate cancer models, as well as a reduction in proliferation and chemo resistance following PARP9 or PARP14 inhibition, in both a STAT-1-dependent and independent manner, respectively [30,55]. This effect was also observed in the reduction of proliferation following PARP9 and/or PARP14 silencing in cultures of different tumors (Figure 2) [30,80].

Specifically, PARP14 silencing in different tumor cell lines led to cell cycle arrest and proliferation inhibition in a manner dependent on the cell cycle protein Cyclin D1, which is overexpressed in various cancer types [81]. This effect on tumor growth reduction was also described in in vivo and in vitro models of hepatocellular carcinoma, along with increased apoptosis and an alteration of the Warburg effect through decreased oxygen consumption and lactate production [81,82]. Furthermore, Wong et al. described that catalytic inhibition of PARP14 with the selective inhibitor RBN012759 restores sensitivity to PD-1 treatment, associated with a decrease in regulatory T cells, enabling sustained tumor regression and generating an antitumor immune memory following co-treatment with PARP14 inhibitor and anti-PD-1 therapy [82,83]. Along with PARP14, PARP15 is also overexpressed in B-cell lymphoma [83]; although efforts have been made to develop more selective inhibitors targeting PARP15, the effect of its inhibition on tumor progression has not yet been described [19,32].

### 3.2. Development of MART Inhibitors. Clinical Applications in Cancer

The design of new and effective PARPi generation with high specificity and a low off-target rate has been a key point of clinical trials in cancer. The number of pharmaceutical companies and laboratories involved in the design, purification, and testing of these molecules has grown exponentially and in parallel with the number of new discoveries about their structure and function in cell biology. Despite this, the clinical effectiveness of PARPi has been limited to the effect on the main nuclear PARPs (DNA-dependent) due to their abundance and relevance in cellular processes [4,20,84]. Selective inhibitors targeting MARTs are very limited, but recent advances in the knowledge of their structure, location, biochemical interactions, knockout models, and described ADP-ribose polymerase activity have led to significant progress in developing MART inhibitors (MARTi) (Table 1) [20]. In this section, we summarize recent advances that have enabled the discovery of inhibitors of MARTs and their applications in cancer progression and tumor biology in a wide range of models, improving the efficacy of immunotherapies by blocking the immunosuppressive activity of regulatory T cells, inducing immune activation in the TME [85], enhancing the efficacy of CAR T cells [85,86,87], or impairing key hallmarks such as metabolic reprograming or aberrant angiogenesis [83]. We must consider that the molecular mechanism by which many MARTs act is due to the effective use of transient silencing models of expression by siRNAs and knockout models (PARP6, PARP8, PARP9, PARP13, PARP15), so the list of MARTi is more limited [20]. Few studies have addressed the role of PARP13 in tumor progression. It has been described that PARP13 depletion increases the expression and protein levels of TRAILR-4, which blocks TRAIL-mediated apoptosis by preventing the binding of this molecule to pro-apoptotic receptors [88]. This suggests that PARP13 has a pro-apoptotic role by preventing the expression of this decoy receptor and inducing TRAIL-mediated apoptosis resistance in various cancer types; however, there is no guaranteed inhibitor that confirms these observations [88,89]. This is not the case for the following MARTs (Table 1).

PARP3 inhibitors: PARP3 is a DNA-dependent PARP, primarily located in the nucleus and centrioles, whose main functions are related to the control of mitosis and DNA repair [56,57]. Dr. Dantzer’s group has made considerable progress in this field, attributing relevant functions to PARP3 and its MART activity in tumor progression and differentiation [56,59]. In recent years, in vitro studies with the PARP3 inhibitor ME0328 (IC_50_: 0.89 μM) have allowed progress in this regard and have provided PARP3 and its chemical control with enormous therapeutic potential. Currently, models of hypoxia-ischemia, heart failure, and muscle development have allowed us to characterize more PARP functions, which are seriously compromised in response to treatment with ME0328 [56,58]. There are not many studies that have demonstrated its effectiveness in vivo in cancer; however, in 2025, Runjie Fan et al. showed that ME0328 alleviated pulmonary inflammation in acute lung injury (ALI) murine models. Mechanistically, ME0328 repressed the PARP3-dependent NF-κB signaling pathway [90], demonstrating that PARP3 MARylates the peptidyl-prolyl cis-trans isomerase A (Ppia), favoring the downstream inflammatory cascade. The therapeutic possibilities of ME0328 must be explored in depth in tumor progression (Table 1).

PARP7 inhibitors: PARP7 is one of the MARTs whose functions are best characterized in tumors. Up to four direct interactions via MARylation have been described, with key regulators acting on pathways of apoptosis, immune response, or malignant transformation [71,74,91]. PARP7 is known to act as a repressor of the type I interferon (IFN-I) pathway, evading the immune response in colon, ovarian, and glioma tumors (Table 1). Furthermore, PARP7 MARylates the transcription factor FRA1, preventing its degradation, thus blocking apoptosis and the immune response. It even modifies, via mADPr, the detoxification protein AHR, the estrogen receptor ER, and even the hypoxia-inducible factor HIF-1α, leading to malignant transformation, adaptation to tumor TME, and metastasis [77,91]. Due to its involvement in cancer, the chemistry and development of PARP7 inhibitors have taken an exponential leap in the last 5 years. The most important example is found with the compound RBN-2397 (Atamparib, IC_50_: <3 nM), developed by Ribon Therapeutics, Inc. RBN-2397 (Atamparib) (ClinicalTrials.gov identifier: NCT04053673). RBN-2397 restores suppressed type I IFN signaling in a broad spectrum of solid tumors. In animal models, treatment with Atamparib completely retracts tumor growth and reactivates the immune response, generating recruitment of macrophages and lymphocytes to tumor niches (Table 1) [71,74]. However, its low bioavailability and high toxicity rate due to its concentration in use >100 mg/kg, have led to its chemical structure being remodeled for several of these parameters. This has resulted in four effective derivatives of Atamparib, many of these are not only effective for PARP7, but also show specificity for other PARPs with Poly ADP ribose transferase activity: (i) I-1 (IC_50_: 7.6 nM), (ii) (S)-XY-05 (IC_50_: 4.5 nM), (iii) Compound 18 (IC_50_: 0.6 nM), and (iv) Compound 8 (IC_50_: 0.11 nM) (Table 1).

Based on their bioavailability and toxicity rate in animal models, the XY-05 derivative and Compound 18 are noteworthy. The reduced bioavailability and high in vivo dose of RBN-2397 led to studies to modify its effectiveness [72,92]. Hong Feng Hu et al. performed structural modifications to reduce the molecular flexibility of RBN-2397. The result was an indazole-7-carboximide derivative called (S)-XY-05 [93]. This PARP7 inhibitor increased the potency and pharmacokinetic properties of RBN-2397, while maintaining the ability to catalytically inhibit PARP7, but increasing in vivo the infiltration capacity of CD8+ T lymphocytes, the antitumor effect became more effective [93]. Compound 18 is an RBN-2397 derivative containing a hexahydropyrazino [1,2-d]pyrido [3,2-b][1,4]oxazine motif, which internally presents fused rings that give it not only specificity but also a very high inhibitory capacity on the active MAR-PARP7 [94]. The proven pharmacokinetic properties and oral bioavailability of Compound 18 make this drug a candidate to successfully complete preclinical studies, surpassing the antitumor effectiveness of the original RBN-2397 (Table 1) [94]. Despite the new studies, other groups have shown that the combined use of RBN-2397 together with ARH pathway agonists (target described for PARP7), shows great effectiveness on various tumor lines of colon, lung, kidney, breast, or melanoma, manifesting a clear mechanism of synthetic lethality; a very promising study and of great clinical relevance [74,95].

There are 3 other independent groups of PARP7 inhibitors, some of which far surpass Atamparib and its derivatives (Table 1). We highlight the following.

(a) KMR-206 and its derivative Phthtal01: All of them have the ability to inhibit PARP7; however, their ambiguity regarding the inhibition of other PARPs, as well as the lack of reliable in vivo data and their high IC_50_ values, greater than 10–12 nM, make KMR-206 molecules unlikely to pass preclinical studies [92].
(b) PAN-PARP inhibitors: Mainly Thioparib (IC_50_ PARP7: 149 nM) and Cpd36 (IC50 PARP7: 0.21 nM). Both inhibitors again proved to be very ineffective in vitro and in animal models, where there is no strong data to support their clinical future in melanoma, colon, or liver tumors [94,96].
(c) Bifunctional Conjugates (B3 and C6): In this case, both conjugates at the concentration at which they show specificity for the catalytic center of PARP7, in in vitro assays, have shown the ability to prioritize the inhibition of PARP1 and PARP2, which means that despite the main target pathways of PARP7 being affected, there is no conclusive data to support its evolution to the preclinical stage (Table 1) [97].

PARP10 inhibitors: PARP10 is presented as an attractive therapeutic target in cancer and neurodegenerative diseases [36] since it regulates cell proliferation through multiple pathways of the β-catenin pathway in addition to playing a regulatory role in replication and in the mitochondrial response to oxidative stress [36,67,98]. Fluorescent probes have been used to characterize the levels of NAD^+^ consumption by PARP10, although deciphering its ADP-ribose activity and characterizing monomers exclusively due to PARP10 remains a challenge [19,84,98].

In 2017, a race began to develop catalytic inhibitors of PARP10 in order to describe their physiological implications in greater detail, as well as to use them in models of neurodegeneration and cancer. The first designs were based on analogs of the 3.4-dihydroisoquinolin-1 (2H)-one (dq) scaffold molecule [99]. A compound called OUL35 (IC_50_: 329 nM) was designed in 2019, exhibiting >12-fold selectivity on PARP10 over other PARP family members and being able to sensitize various cell lines to treatment with hydroxyurea [19]. Subsequent studies have demonstrated that OUL35 also exhibits affinity for the catalytic centers of other MARTs such as PARP15 and PARP14, a result of special therapeutic interest [83,100]. Years later, ring modifications were introduced into OUL35, specifically cycloalkyl (8a–c), o-fluorophenyl (8h), and thiophene (8l) rings, which allowed the IC_50_ to reach values of 130 to 160 nM (Table 1). These new products have further increased their specificity for other tumor-targeting MARTs, such as PARP14 and PARP15 [32,101]. More recently, molecular dynamics simulations and virtual modeling tests have shed new light on the characterization of new PARP10 molecules, considering a pharmacokinetic criterion, the molecule pharmacophore-based virtual screening was performed on ZINC and NCI libraries has identified that the compound ZINC20906412 offers high therapeutic potential in cancer, although these studies need to be further developed by performing extensive testing in cell models [102].

PARP11 inhibitors: PARP11 is one of the most innovative regulators of the evasion of antitumor immunity and resistance to therapies in solid tumors, playing key roles in the immunosuppressive tumor microenvironment (TME) [87]. In 2018, Kirby et al. developed specific inhibitors of PARP-11 by taking advantage of structural differences between the active sites of DNA-dependent PARPs and active MARTs with described MARylation activity [85]. This study demonstrates that the molecule ITK7 (IC_50_: 14 nM) inhibits PARP11 auto-MARylation, dissociating the enzyme from the nuclear envelope, where PARP11 colocalizes with the nuclear pore and modifies key proteins involved in the organization of nuclear pores, discovering a new promising pathway to explore in human diseases (Table 1) [85,87].

PARP14 and PARP16 inhibitors: The effect of inhibiting PARP14 on metabolic reprogramming phenomena in tumor models in response to energy stress. RBN012759 (IC_50_: <3 nM), the first highly potent PARP14 inhibitor, was purified in 2018, enabling it to reverse IL-4-driven gene expression in macrophages induced by TME factors, mainly affecting the pro-inflammatory cascade associated with kidney cancer tumor explants [103]. PARP16 localizes to the endoplasmic reticulum and modifies mediators of ER stress responses (UPR in many scenarios), such as PERK and IRE1, where it acts as a potent inhibitor of protein translation by catalyzing mono-ADP-ribosylation of ribosomal subunits, such as RPL14 and RPS6, thereby inhibiting polysome assembly and mRNA loading [104,105]. RBN010860, a pan-MART inhibitor that binds to H-Y-I/L/Y PARPs (IC_50_: <0.1 μM), is considered a potent PARP16 inhibitor, modulator of the ER response to oxidative stress of mitochondrial origin. Like RBN-2397 derivatives, its adequate druggability means that it is currently in the preclinical phase (Table 1) [10,106].

In conclusion, given the key role of MARTs in cellular function and their involvement in the development of diseases such as cancer, there is a pressing need to deepen our understanding of MARTs and MARylation. This can be achieved through the development of innovative strategies to study their biological targets and the search for more specific inhibitors that can mimic the tumor-suppressive effects of MART silencing in cancer progression.

## 4. Poly ADP-Ribose Glycohydrolases and Other PAR Erasers. Implications for Cell Fitness

Modifications by PARylation are considered key events for the maintenance of DNA architecture and biochemistry. Considering the wide variety of structural proteins, kinases, or repair enzymes that can be substrates of poly ADP-ribose (pADPr), the ADP-ribosylation cycle must be controlled at several levels. The selective removal of ADP-ribose polymers is carried out by poly(ADP-ribose) glycohydrolase proteins (PARGs) [4,107,108]. PARG enzymes may have a protective effect against excessive PARP engagement; on the contrary, it is described that the catalytic inhibition of PARG prolongs the levels of ADP-ribosylation on the points of DNA damage induced by chemotherapeutics, triggering cell death in certain cancer models with defects in DNA repair systems, through synthetic lethality [109]. PARG acts as an endo-glycohydrolase and an exo-glycohydrolase, hydrolyzing glycosidic bonds, producing free PAR and mono ADP-ribose moieties, respectively [11]. The free ADP-ribose is then metabolized into AMP and ribose 5′-phosphate residues by Pyro-phosphohydrolases such as NUDIX family enzymes [6]. Finally, AMP is utilized as a key metabolite in ATP synthesis, lipid metabolism, and cell signaling messenger in Ca^2+^ pathways [48,110]; ribose 5′-phosphate moieties will represent a key precursor to many biomolecules [48,111]. On the contrary, PARG cannot remove the terminal ADP-ribose or those modifications based on mono-ADP ribose or MARylation. These latter residues require a set of “additional hydrolases” such as terminal ADP-ribose glycohydrolase (TARG1), or ADP-ribose-acceptor hydrolases ARH1/3 [112,113]. Recent studies have demonstrated several terminal ADP-ribose eraser functions in other macro domain-containing proteins, such as hMacroD1/D2 [4,114].

**Figure 3:**
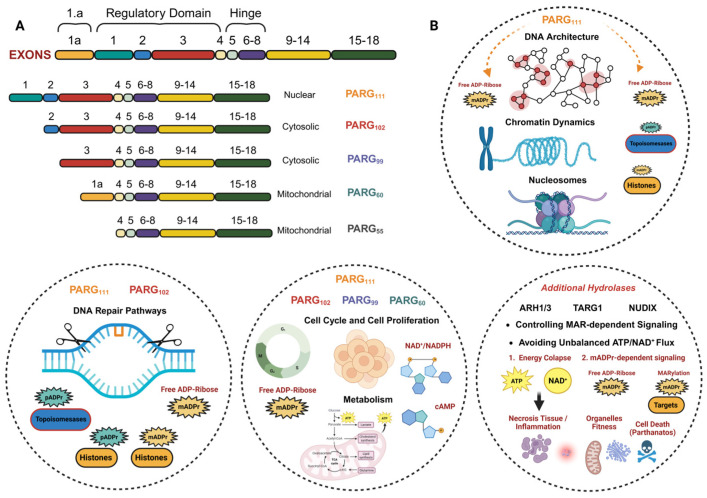
Overview of PARG isoforms and their functions in cells: (**A**) Structure and cellular location of the 5 PARG isoforms, derived from mRNA splicing of the original gene. (**B**) Main functions of PARG isoforms. PARG111, as a nuclear isoform, along with PARP102, plays a key role in maintaining the stability of the cellular genome and related functions. The cytosolic isoforms (PARG102, PARG99, and PARG60), together with PARG111, are responsible for maintaining cell cycle progression and metabolic homeostasis. PARG50, as a mitochondrial protein, has fewer described functions, although it is associated with mitochondrial dynamics and function. Additional hydrolases help complete the ADP-ribose cycle, preventing energy and reducing power collapses, as well as a lack of second messengers. Their implications for cell fitness and cellular adaptation are very important, which is why they are also considered clinically relevant. (**A**) modified from Harrision, D. et al. (2020) Sec. Cellular Biochemistry. (**B**) Created in BioRender. Rodríguez Vargas, J.M. (2025) https://BioRender.com/0np0g0w (accessed on 10 December 2025).

PARG translation is encoded from a single gene (18 exons) codifying for a full protein consisting of a regulatory domain at the N-terminus and the catalytic domain at the C-terminus (Figure 3A). The conserved catalytic domain is maintained together with various splicing events that guide the translation of 5 PARG isoforms, which localize to different cellular compartments [13,108] (Figure 3A). PARG possesses at least 4 potential nuclear localization sequences (NLS), 3 nuclear export signals (NES), and at least 1 mitochondrial targeting sequence (MTS) [11]. However, the physiological role and clinical relevance of the isoforms are far from clarified [11,107].

Isoform 111 (PARG111): Full PARG protein; mainly localizes in the nucleus. PARG111 has endo and exo-glycohydrolase activity on ADP-ribose polymers and oligomers, catalytically bound to proteins involved in replication, repair, cell cycle, and DNA architecture [104].

Isoform 102 (PARG102): Cytosolic endo/exo-glycohydrolase implicated in the regulation of miRNA stability, formation of stress granules, and detoxification of unfolded protein [115].

Isoform 99 (PARG99): The 99 kDa isoform localizes in the cytoplasm and maintains its endo-glycohydrolase activity. It removes cytoplasmic poly(ADP-ribose) chains, which helps to regulate cellular processes like protein aggregation, unfolded protein response (UPR), and stress granule response [104,115].

Isoform 60 and 56 (PARG60 and PARG56): Mitochondrial PARG isoforms lacking in activity glycohydrolase. Due to its location and numerous studies linking an active role to free, non-covalently bound ADP-ribose oligomers in the mitochondria, it is attributed a crucial role in mitochondrial dynamics and stability; however, there is no conclusive data on this matter [115,116,117] (Figure 3B).

Despite having 5 isoforms, PARG is found in low cellular abundance; however, in the last 10 years, considerable progress has been made in the therapeutic characterization of each PARG isoform [13,105,118]. The mammalian 39-kD ADP-ribose hydrolase-like protein, or ARH3, also possesses the catalytic activity of pADPr glycohydrolase, although at a very low level, and its contribution to pADPr turnover is not well known [119].

### 4.1. Biological Functions of PARGs and Other Additional pADPr Hydrolases

PARGs are basically ADP ribose-erase proteins; however, like PARP enzymes, PARGs are involved in DNA metabolism, so that their inhibition or elimination sensitizes cells to various treatments, which is considered the molecular basis for their therapeutic impact. By removing pADPr, PARG is critical for processes such as DNA damage repair, chromatin dynamics, transcriptional regulation, and cell proliferation [107,120]. However, the biological role of PARG isoforms remains less understood than the role of PAR synthesis by PARP enzymes. One of the main difficulties lies in the fact that the chemistry of synthesis of effective inhibitors dictates much of achieving high efficacy, to which must be added the fact that there are several isoforms of PARG and the almost impossibility of obtaining viable PARG-null animals. PARG maintains proper pADPr levels, preventing excessive PARP activation and ensuring the efficient progression of cellular processes, so the mechanism of cellular sensitization by attacking PARG activity is based on a process of synthetic lethality [105,114]. The function of PARG in DNA repair is crucial, since it is responsible for eliminating modifications by poly ADP-ribose (PAR) on enzymes responsible for recognizing, repairing, and relaxing the chromatin structure, therefore altering its activity, leading to lethal repair failures that trigger cell death or, where appropriate, mutations that promote malignancy [12,105]. In relation to chromatin architecture, the removal of pADPr and PAR modifications by PARG influences histone localization, nucleosome organization, and chromatin decondensation, which is essential for the regulation of gene expression and other nuclear processes such as replication or transposition of DNA sequences (transposons) to different locations within a genome [7,121,122] (Figure 3B).

Recent studies using both in vitro and in vivo models have shown that controlling pADPr levels by PARG through its inhibition, silencing, or knockout modulates cell cycle progression and enhances the proliferation rate. PARG facilitates cell transformation and invasion in several tumor models, establishing that the DePARylation levels are required in S phase progress [123,124], so much so that its inhibition leads to cycle arrest and cell death. The molecular mechanism by which PARG controls this step requires further study in order to identify PARP1-mediated PARylation and PARG-catalyzed DePARylation targets in S-phase progression, applying proteins involved in Okazaki fragment ligation and/or base excision repair, regulated pADPr signaling, and PARG signaling [123]. Multiple tumor models that manifest resistance to PARP inhibition (PARPi) showed metabolic reprogramming through the increased availability of NAD^+^. In this scenario, specific PARG inhibition (PARGi) in combination with cell cycle checkpoints abolishment, exhibited excessive replication stress-mediated DNA lesions, cell cycle dysregulation, and mitotic catastrophe in PARPi-resistant cancer cells, highlighting a novel crosstalk between metabolism, DNA integrity, and NAD^+^-dependent PARylation [125,126]. In conclusion, PARG plays a critical role in tightly controlling the levels of PAR produced during genotoxic stress to prevent the detrimental effects of PAR overaccumulation, offering enormous therapeutic potential in human diseases such as cancer, mitochondrial disorders, and neurodegenerative syndromes, where the balance between metabolism, PARylation, and DNA stability is a major factor (Figure 3B).

Whereas the main poly-ADP-ribosylation erasers are relatively well described, the enzymes involved in mono-ADP-ribosylation (MARylation) recycling have been less well investigated. In this way, mono(ADP-ribose) (mADPr) hydrolases such as the ARH and NUDIX family or the terminal ADP-ribose glycohydrolase (TARG1) require further study in order to identify their function and location, as well as their implications in biological processes. ADP-ribosyl-acceptor hydrolases (ARH) constitute a family of 3-member enzymes (ARH1-3) with a size (39 kDa) and amino acid sequence very similar to each other [127]. ARH1 catalyzes the hydrolysis of the N-glycosidic bond of mono-(ADP-ribosyl) ated arginine in several targets implicated in DNA repair, toxin response, and carcinogenesis [112,127,128]. ARH3 hydrolyzes poly-(ADP-ribose) (PAR), manifesting PARG activity, and O-acetyl-ADP-ribose, avoiding ADP-ribose-arginine, -cysteine, or -asparagine bonds. The 39-kDa ARH3 shares amino acid sequence identity with both ARH1 and the catalytic domain of PARG. ARH3 participates in the degradation of PAR that is synthesized by PARP1 in response to oxidative stress-induced DNA damage; this hydrolytic reaction suppresses PAR-mediated cell death, a pathway termed parthanatos (Figure 3B) [112,129].

NUDIX (Nucleoside Diphosphate-X) hydrolases are proteins involved in NAD^+^ metabolism, cellular homeostasis, mRNA transcription and maturation, and the recycling of ADP-ribose residues [130,131]. Their involvement in PARP/PARG/mADPr metabolism makes them proteins of current therapeutic interest in metabolic diseases such as cancer or neurodegenerative diseases, thanks to their involvement in mitochondrial stability and function. pADPr (PAR) is synthesized by PARPs in response to DNA damage to signal for repair, while NUDIX hydrolases, including enzymes like NUDT5 and NUDT16, can degrade free or protein-conjugated ADP-ribose, with some also playing roles in the energy derangement during high PARP activity by producing AMP [132], preventing PARP over activation and energy collapse due to high ATP and NAD^+^ consumption [131,133]. In conclusion, it could be linked to indirect regulation of cell death processes such as apoptosis and parthanatos, and therefore to tumor resistance. Specific human NUDT16 can cleave PAR and mono(ADP-ribose) (MAR) directly from proteins, finely regulating the accumulation of AMP and mitochondrial dynamics in normal and pathological situations (Figure 3B) [133].

TARG1 is an ADP-ribosylhydrolase that removes aspartate/glutamate-linked ADP-ribosylation [134]. TARG1 plays a key role in recognizing and DNA repair [135]. Loss of TARG1 sensitizes cancer cells to inhibitors of topoisomerase II and PARP inhibitors, triggering a synthetic lethality mechanism driven by a toxic accumulation of mADPr that induces replication stress and genomic instability [135]. Moreover, several studies have demonstrated that telomere replication and integrity are strongly connected to TARG1 activity on telomere pADPr catalyzed by PARP-1, highlighting its potential role in cancer cell signaling and survival (Figure 3B) [136].

### 4.2. Targeting DePARylation in Cancer Therapy. Potential Therapeutic Opportunities

**Figure 4:**
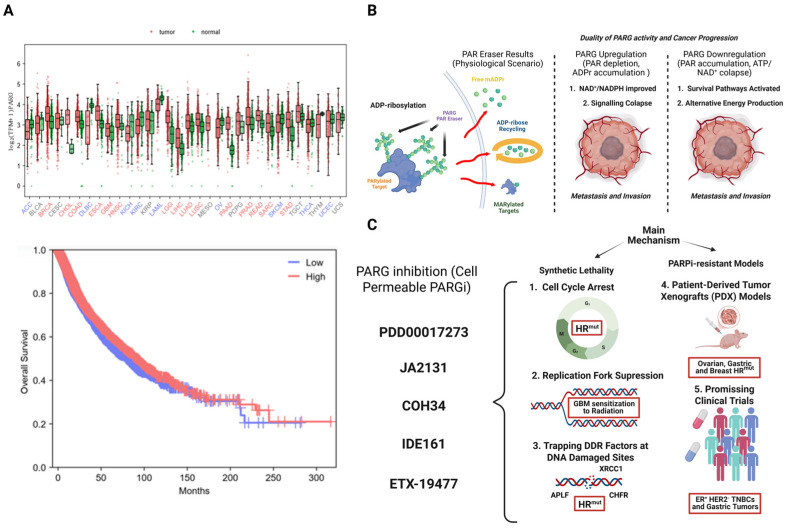
PARG druggability, tumor relevance, and clinical development stage: (**A**) Compared expression profiles for PARG between tumor versus normal tissues using TCGA Pan-Cancer Atlas data analysis obtained from GEPIA3 (Gene Expression Profiling Interactive Analysis). Down showing Kaplan–Meier survival plots for PARG. Overall survival in cancer patients with low or high PARG expression. (**B**) Duality of PARG activity in cancer progression. Dysregulation of PARG activity has severe consequences for both non-malignant and tumor cells, directly impacting metabolism, genome instability, and cell proliferation. In cancer, PARG overactivity promotes signaling collapse, which, for example, in scenarios like tissue hypoxia, favors malignancy and metastasis. Conversely, and far from being harmful, a decrease in PARG activity causes an energy and NAD^+^/NAPH collapse, favoring “alternative malignancy pathways” such as autophagy. (**C**) Overview of the main cell-permeable PARGi and its clinical implications in cancer. Two main action mechanisms are described, based on synthetic lethality models, associated with HR mutations and replication fork suppression, and based on PARPi resistance scenarios, in which case, the results obtained show a very promising future for patients with this type of resistance. Compare data with Table 2, which includes information on the clinical status of PARGi. (**A**) Obtained from clinical data, http://gepia.cancer-pku.cn/ and https://gdc.cancer.gov/about-data/publications/pancanatlas, (accessed on 10 November 2025). (**B**,**C**) Created in BioRender. Rodríguez Vargas, J.M. (2025) https://BioRender.com/ypmjoxk (accessed on 10 November 2025).

Inside the PARP family, a large number of members are considered prognostic markers in various types of tumors. Data extracted from several databases, such as cBioPortal and the TCGA, demonstrate that PARP1, PARP3, PARP6, PARP7, PARP10, and PARP12 are overexpressed in various solid tumors, such as rectum adenocarcinoma (REAC), grade IV glioblastoma (GBM), breast cancer (TNBC), or mesothelioma (MESO); their expression is associated with malignancy and metastasis. According to our analysis in the TCGA database, a large number of tumors overexpress PARG. For this review, we compared expression profiles for PARG between tumor versus normal tissues using the TCGA Pan-Cancer Atlas and obtained data analysis from GEPIA3. We found that tumors such as bladder urothelial carcinoma (BLCA), cervical squamous cell carcinoma and endocervical adenocarcinoma (CESC), esophageal cancer (ESCA), grade IV glioblastoma (GBM); cervical kidney renal papillary cell carcinoma (KIRP), sarcoma (SARC), and thymic carcinoma (THYM) showed upregulation of PARG, while in adrenocortical carcinoma (ACC), diffuse large B cell lymphoma (DLBC), acute myeloid leukemia (LAML), ovarian tumor (OV), and thyroid carcinoma (THCA), PARG isoforms are downregulated. In both cases, all our data indicated the high clinical relevance of PARG and its activity on ADP-ribose metabolism in cancer (https://www.cancer.gov/ccg/research/genome-sequencing/tcga (accessed on 10 November 2025) (Figure 4A). All data were contrasted using https://gdc.cancer.gov/about-data/publications/pancanatlas (accessed on 10 November 2025). We also performed Kaplan–Meier survival plots for PARG, showing overall survival curves in cancer patients with low and high PARG expression (data analysis and graphs from GEPIA3, *p*-value 0.0151). We concluded that there is a fairly clear positive correlation between high levels of PARG and the drop in survival and life expectancy of patients (http://gepia.cancer-pku.cn/ (accessed on 10 November 2025) (Figure 4A).

The importance of adequate levels of pADPr and mADPr for maintaining cellular homeostasis has been established for decades. PARP1 is capable of synthesizing up to 95% of all ADP-ribose polymers necessary to maintain DNA integrity, regulate gene expression/repression levels, and modulate genome architecture and various cytoplasmic proteins. Considering that genomic instability derived from deficiencies in DNA repair and architecture contributes to the appearance and progression of tumors, the clinical relevance of controlling ADP-ribosylation levels is one of the main objectives on which antitumor therapies are focused [4]. Based on this, recent studies are demonstrating that therapeutic disruption of ADP-ribosylation/DePARylation processes selectively kills cancer cells or at least slows and conditions tumor progression, rendering them susceptible to complementary treatments (Figure 4B) [11]. The chemical development of both PARPi and PARGi (or PARG inhibitors) is becoming increasingly important, and increasing amounts of experimental resources are being devoted to obtaining clinically effective molecules. The removal of pADPr chains is mainly attained due to the hydrolysis of these polymers by PARG isoforms [11]. However, since PARG cannot eliminate terminal ADPr, it directly intervenes in the regulation of a multitude of important pathways and processes such as differentiation, inflammation, and tissue regeneration (Figure 3B) [137,138]. In certain tissues such as the heart, lung, and brain, it has been shown that the remaining ADPr residues and even the monomers and small oligomers bound to targets trigger adaptation and recovery mechanisms following episodes of hypoxia, ischemia, reperfusion, or acute respiratory failure [139,140].

In cancer, the abnormal and uncontrolled growth, as well as an insatiable capacity to monopolize resources, are the two main morphological and biochemical characteristics that have received the most research. However, focusing new projects on other acquired hallmarks, such as angiogenesis, immune evasion, programmed cell death, or immune evasion, presents a broad field of study that continues to advance. PARPi have traditionally been shown to be efficient in a wide range of solid tumors with certain genetic characteristics; in this new section, we will explore the main therapeutic advances derived from inhibiting or blocking DePARylation pathways in cancer progression (Figure 4B).

#### 4.2.1. Developing PARG Inhibitors (PARGi) as a Novel Cancer Therapy

DePARylation is not merely an antagonist pathway of PARylation in the context of DNA repair. DePARylation is an immediately downstream step of PARP-dependent DNA repair; despite this, it must be considered that its functions related to chromosomal architecture and stability make DePARylation a target of great clinical interest, as well as a critical pathway for cancer cells’ viability [141].

Immediate Consequences for tumor cells (Metabolism and DNA Fitness): By preventing the proper removal of pADPr from signaling proteins (PARP), cells relax chromosome strands (topoisomerases and histones) and repair damaged DNA (DDR) less efficiently, compromising SSB and DSB repair pathways [141]. The consequences of cancer could be significant, including accumulating genomic instability or deregulating cell survival and death pathways. Moreover, PARG inhibition impairs replication fork dynamics, leading to reduced fork progression, accumulating DSBs, and ultimately leading to replication catastrophe and cell death [108,124]. In metabolism, inhibiting PARG activity has major consequences; the balance in the ADP-ribose cycle depends on the equilibrium between NAD^+^ consumption and its recycling to AMP in order to produce new biomolecules. Excessive PARP or PARG activity, as well as a blockage of one or both, compromises this cycle and leads to two immediate consequences: (a) energy collapse due to excessive ATP/NAD^+^ consumption or ADPr accumulation, and (b) signaling collapse due to an imbalance in the NAD^+^/cAMP ratio, as key signaling molecules implicated in cell cycle, proliferation, cell death, and metastasis (Figure 4B) [137].

Long-term consequences for tumor cells (PARPi Resistance): Considering the mechanism of action, it is feasible to contemplate PARGi as a clinical alternative of great power in models of resistance to treatment with PARPi, even more so when the possibility of synthetic lethality between PARPi and PARGi is studied in cancer. The increased, and sometimes excessive, use of PARPi such as Olaparib, Rucaparib, or Niraparib, on various types of tumors, including some that do not manifest HR deficiencies, has led to the appearance of many cases of resistance in monotherapy [142]. The main mechanisms of resistance to PARPi are (i) restoration of the HR capacity, mainly occurring in response to a reversal of the BRCA1/2 mutation; (ii) mutations in PARG111, or (iii) stabilization of the replication forks. PARPi triggers PARP1 trapping at the replication fork and double-strand breaks (DSBs), repairable by HR repair. Mutations in PARG111 promote the accumulation of pADPr in DNA-dependent PARPs and maintain auto-PARylation of PARP1; consequently, the final rescue of PARP1 from the damage forks occurs, triggering resistance. Currently, several alternatives are being considered to counteract resistance to PARPi treatments, including immunotherapy, combined therapies with CDK inhibitors, MAP kinase inhibitors, and anti-angiogenic drugs. One of the most promising therapeutic possibilities is to directly attack the genome stability of the tumor cell and the metabolic reprogramming intrinsic to the processes of malignancy, adaptation to TME, and metastasis, through the use of PARGi in order to sensitize PARPi-resistant cells to subsequent radio or chemotherapy treatments or to generate PARPi/PARGi synthetic lethal models (Figure 4B) [115].

Next, we will explore the evolution of molecules capable of inhibiting PARG, blocking DePARylation, their pharmacological properties, and their effectiveness in treating tumors. In the cases that will be presented, the main objective of treatment with these molecules is to trap DNA repair factors on the pADPr chains, thus suppressing DNA damage repair and inducing cell lethality (Table 2 and Figure 4B).

DNA Intercalating Molecules: The first generations of PARG inhibitors were described as DNA intercalating polyaromatic molecules such as Proflavine, Ethidium Bromide, and Ethacridine. These inhibitors specifically bind the ADPr recognition site of PARG, inhibiting PARG-mediated pADPr hydrolysis, and introducing genome instability and DSBs [143,144]. Since this type of damage is a clear target of HR, it would result in a specific mechanism of attack on proliferation and viability, or at least on a possible sensitization of tumor cells. These drugs have proven to be highly nonspecific and ineffective, which has led to high doses in vitro or ex vivo, preventing their effective use in animal models. Furthermore, the high rate of toxicity and the induction of genomic instability mean that intercalating agents have off-target effects on tumor cells and non-transformed cells [141].

Tannins (Nobotanin K) (IC_50_: ≥0.3 μM): Natural tannin is soluble in water and purified from the leaf extracts of several plant species. Its mechanism of action is based on acting as an analog of ADPr residues, allowing it to bind to ADP ribose (PAR) chains, inhibiting the endo- and exo-glycohydrolase activity of PARG111 and PARG102 isoforms. Although its druggability is moderate, it is not very selective, it is very impermeable to cell membranes, and its high doses have made it difficult to obtain reliable in vivo data [109].

GPI-16551/GPI-18214 (IC_50_: ≥2 μM): Both molecules block the pADPr hydrolysis catalyzed by nuclear and cytoplasmic PARG. GPIs exhibit low affinity and ex vivo effectiveness on PARGs. Their high effective doses, coupled with numerous cell permeability issues, make it difficult for both molecules to obtain consistent results across different research groups. Even so, GPIs are in the preclinical phase and are expected to have a promising therapeutic future.

ADP-HPD (adenosine diphosphate-(hydroxymethyl) pyrrolidinediol) (IC_50_: 0.12 μM): ADP-HPD is a chemical analogue of ADP ribose, therefore preventing PARG activity on PAR polymers and oligomers. Although highly selective, its druggability is very low. The main limitation to its use is its inability to penetrate cell membranes in several cancer cell models, which limits its effectiveness to in vitro trials. ADP-HPD is in a purely experimental clinical stage, and its synthesis chemistry has been revised on numerous occasions [115,137].

Rhodamine-Based PARG inhibitors (RBPIs) (IC_50_: 1–6 μM): RBPIs are considered small, highly selective molecules with a strong affinity for the active site of PARG. Despite numerous examples of cellular impermeability or difficulty in incorporating them, they are considered to have few limitations in their pharmacological applications against various solid tumors, which consolidates their position as one of the leading drugs of choice. Currently, oral administrations are being tested in animals using xenograph models of breast, colon, and melanoma with relative success [137] (Table 2).

In the next section, we will explain how treatments with the newest and most pharmacologically active PARGi can be used to induce synthetic lethality mechanisms, blocking treatment resistance events in PARPi-treated cancer. In order to achieve greater specificity and efficacy while minimizing off-target effects, the chemical design and screening were geared toward obtaining synthetically lethal molecules. As with most PARPi, the next studies were performed on tumor models with defects in DNA repair (HR in particular) and alterations in replication and/or transcription.

#### 4.2.2. Synthetic Lethality Is a Response to PARGi in Cancer Cells

In the previous section, we described how PARPi-dependent resistance primarily occurs. Over the last five years, efforts have been made to reverse and re-sensitize tumor cells using a reverse methodology. This involves harnessing the ability of tumor cells to develop resistance in response to PARPi and turning it against them. This new strategy for targeting acquired vulnerabilities will be based on treating these tumor models with PARGi. Despite their proven efficacy in vitro and ex vivo, the vast majority of PARGi are still far from being effective in clinical treatment; however, there is a group whose chemical formulation, efficacy, and druggability allow them to be in preclinical stages and even in phase I and II trials. It has been established that the most efficient combinations of treatments in response to PARP inhibition resistance are PARPi + anti-angiogenic agents, PARPi + immunotherapy, PARPi + PI3K/AKT pathway inhibitors, PARPi + Ras/Raf/MEK/MAPK pathway inhibitors, and PARPi + epigenetic modifiers [118].

Although preliminary pharmacokinetic data have been promising, the short duration of the effective response in tumors and the toxicity profiles suggest that the effectiveness of these therapies is not yet as expected; even so, these types of therapies are in the preclinical phase or even, in some cases, in phase I. It is here that synthetic lethality models PARPi/PARGi are gaining interest from the clinical community (Figure 4C).

Below are the most obvious examples of effectiveness within the broad spectrum of PARGi.

PDD00017273 (IC50: 26 nM): Potent and selective inhibitor of PARG endo-/exo-glycohydrolase activity of PARG, tested in purified PARG111, PARG102, and PARG99 isoforms and total protein extracts, with an IC50 of 26 nM in in vitro PARylation enzyme assays and 37 nM in cells [145]. PDD000117273 has even been shown to be effective in inhibiting ARH3 activity, which marked an important milestone in cell physiology, due to the importance of maintaining MARylated targets involved in cell proliferation or metabolic adaptation [145,146]. Its pharmacology indicates a short effective half-life, which makes its in vivo use relatively restricted; however, it has proven very efficient in triggering synthetic lethality in models with compromised HR in BRCA1/2, PALB2, or BARD1 breast, colon, ovarian, and pancreatic ductal adenocarcinoma [145].

JA2131 (IC_50_: 0.4 μM): A small 6′-thio analog of a methyl guanine derivative, producing an effective PARG inhibition. The tumor relevance of JA2131 is based on causing replication fork stalling and apoptotic cell death. JA2131 exhibits excellent cell membrane permeability. This particularity allows it to be effective in tumors with marked drug elimination systems, such as ABC pumps, and detoxification systems via autophagy or secretory vacuoles; it is effective in GBM-type tumors, where synthetic lethality is beginning to be described with PTEN, which is mutated in a large proportion of these tumors, and with IDH1, whose isogenic mutation is induced to study resistance and invasion mechanisms in grade IV astrocytomas [126,147].

COH34 (IC_50_: 0.37 nM): Potent and specific PARG inhibitor, binding to the catalytic domain, suppressing glycohydrolase activities. Prolonged PARylation induced by COH34 triggers DNA repair factors, such as XRCC1, APLF, and CHFR at the DNA-damaged sites, exhibiting synthetic lethality in HR compromised breast (TNBCs and basal B tumors) and ovarian cancer cells, as well as in hepatocellular carcinoma [109,148].

PDD000117273 Derivatives: (i) IDE161 (IC_50_: not publicly available): A derivative of PDD00017273 developed by IDEAYA Biosciences, recently approved for use in patients by the U.S. Food and Drug Administration, currently in phase I/II clinical trials for various cancers with homologous recombination defects (ovarian, breast, or gastric cancer) [137]. (ii) ETX-19477 (IC_50_: not publicly available) is an orally bioavailable small molecule inhibitor of PARG, developed by 858 Therapeutics. ETX-19477 attenuates the DNA repair process and induces cell cycle arrest and mitotic catastrophe, leading to programmed cell death (apoptosis) in ER+ HER2- TNBCs, mucinous ovarian cancer, lung, and gastric cancer (Figure 4C) [137,149].

The most recent studies are based on the exclusive development of targeted drugs for PARPi-resistant tumors, based on the premise that PARG inhibition increases PARylation levels and may prevent dissociation of PARP1 and PAR-binding repair proteins (e.g., XRCC1) from DNA-damaged sites. Loss of PARG activity causes fork stalling and prevents the restart of reversed forks. In this way, PARP inhibitors used in cancer therapy (Olaparib, Rucaparib, Niraparib, and Talazoparib) as predictive biomarkers of inhibitor sensitivity, are being analyzed in models where PARylation is maintained in a non-physiological way in HR-deficient cancers. PARPi-resistant phenotypes acquired by these cells are analyzed in detail, understanding the molecular consequences of maintaining PARylated proteins such as guardians of the genome, regulators of epigenetic profiles, and DNA repair enzymes [11,141]. Biochemically, the combination of PARG inhibition and PARPi resistance models focus on basic aspects of the tumor, such as mitochondrial dynamics, ER/mitochondria interactions, and the use of lipids as fuel to adapt to oxidative stress or anaerobic glycolysis. This advancement offers a novel therapeutic approach for cancer patients (Figure 4B,C and Table 2).

## 5. Future Perspectives

PARP proteins and the ADP-ribosylation process play an essential role in a multitude of physiological processes, while their deregulation in terms of gene expression or activity attributes to them a multitude of actions in diverse human pathologies, ranging from metabolic and neurodegenerative diseases to cancer.

Therapeutic targeting of PARylation and DePARylation represents an important strategy in precision cancer medicine. The development of PARPi has allowed to precisely characterize therapeutic targets in various cancer models, in which the strategy has been focused on the DNA damage response and stability of the replication fork. Based on a synthetic lethality model, PARPi allow for precise effect against the progression of tumors with a specific genetic background. However, the spectrum of tumors sensitive or sensitizable to PARPi goes beyond the presence of a prominent or deficient homologous recombination repair system. The main problem with PARPi lies in the fact that although PARPi have a significant clinical importance in various tumor types, including certain metabolic diseases, PARPi induce high rates of adaptation and resistance in tumors, even in those with mutations that are likely to generate synthetic lethality on p53 or BRCA1. This emergence of PARPi-resistant cancers has prioritized in recent years the development of alternative therapeutics, targeting PARylation readers and erasers. DePARylation, a sequential downstream pathway to PARylation, holds significant potential as a therapeutic target.

The molecular mechanism by which PARylation controls replication and transcription, as well as triggers or represses downstream metabolism, requires further exploration. These new discoveries open the door to new lines of research on how maintaining ADPr (mADPr and pADPr) levels negatively influence cellular fitness. Cancer is capable of triggering metabolic reprogramming pathways, which allow it to transport lipids, prioritize anaerobic glycolysis, or modulate mitochondrial dynamics processes. All of these are highly dependent on available NAD^+^ levels. The NAD^+^ pool will largely depend on the ADP-ribose cycle and the balance between ADPr writers and erasers. Inhibiting PARPs strengthens NAD^+^ levels, allowing for the promotion of adaptive pathways such as autophagy, glycolysis, or beta-oxidation. However, it has been confirmed that the therapeutic use of PARGi counteracts this process and consequently favors the sensitization of tumor cells in two main ways: **(a)** directly attacking the replication folks by the effect of PARGi on PARP activity and other guardians of the genome and **(b)** compromising the metabolic response preferably at the mitochondrial level, favoring processes of programmed cell death or sensitization to recommended chemotherapeutic agents.

Additionally, other DePARylation enzymes (e.g., TARG1 and ARH3) are also known to be involved in human diseases, such as neurodegenerative disorders and cancer. Thus, the mitigation of these debilitating diseases should be further investigated when the PAR metabolism is targeted. Recent studies provide interesting data on targeting other erasers, but molecular and in vivo application constraints make it difficult to obtain significant therapeutic data.

MARylation and MART enzymes control a multitude of cellular events in cancer, both in mono-regulatory mechanisms based on mono ADP-ribosylation, such as inflammatory response, DNA replication, or transcription, as well as compensation phenomena with other PARPs, mainly tankyrases and DNA-dependent PARPs, which attribute a role to them in progression and adaptation to the TME (hypoxia, starvation, or EMT). Furthermore, new strategies of MARylation detection and the current state of MARTs inhibitors were discussed previously in this manuscript, so we highlight an outlook for future study, aiming to reveal the unknown biological properties of MARylation and its relevant mechanisms, and establish a novel therapeutic perspective in human diseases.

In conclusion, the global study of all the players involved in the ADP-ribose cycle presents a promising future for the identification of new targets in cancer. Considering the need for increasingly personalized medicine based on immunological treatments, the combination of omics data with in vitro and ex vivo assays will provide the molecular basis for the vast majority of ADP-r writers and erasers to become clinical targets in tumor progression.

## 6. Conclusions

The poly(ADP-ribose) polymerase (PARP) family, comprising 17 members, represents a major group of proteins involved in the maintenance of cellular homeostasis at multiple levels. While DNA-dependent PARPs are primarily associated with genome stability, other family members, such as tankyrases and several mono-ADP-ribosyltransferases (MARTs), play important roles in DNA transcription, replication, and repair. Through poly-ADP-ribosylation (PARylation), mono-ADP-ribosylation (MARylation), and non-covalent interactions mediated by ADP-ribose residues, PARPs regulate a wide range of cellular processes, including cell cycle progression, cell death and differentiation, inflammatory signaling, host defense responses to pathogens, cell survival pathways (such as autophagy, the unfolded protein response, and oxidative stress detoxification), and cellular metabolism.

The clinical relevance of PARPs and ADP-ribosylation metabolism has increased substantially following the development of PARP inhibitors (PARPi, FDA-approved). Inhibition of ADP-ribosylation directly suppresses PARP enzymatic activity, thereby preventing protein, RNA, and DNA modifications and their downstream functional consequences. However, PARP inhibition also induces profound metabolic alterations, as cellular ATP, NAD^+^, and cAMP levels are largely influenced by PARP-mediated substrate consumption for ADP-ribose synthesis, as well as by increased NAD^+^ availability for competing enzymes, such as sirtuins. Consequently, cellular metabolism shifts toward altered glycolytic flux, fatty acid β-oxidation, and protein synthesis. In the context of cancer, this metabolic reprogramming is a critical determinant of tumor progression and responsiveness to antineoplastic therapies. Accordingly, imbalances in the ADP-ribose cycle—arising from PARP overactivation, PARP inhibition, or dysregulated ADP-ribose recycling (DePARylation)—create unfavorable conditions for most cell types.

Numerous PARP inhibitors have demonstrated clinical efficacy; however, an increasing number of resistance cases have been reported in specific tumor types. In general, the therapeutic rationale of PARP inhibitors relies on synthetic lethality associated with tumor-acquired mutations, most commonly defects in homologous recombination (HR) repair pathways, such as BRCA1/2 mutations. Agents including Olaparib and Rucaparib have shown clinical benefit in breast, ovarian, prostate, and melanoma cancers. Both are currently approved or under evaluation across multiple treatment settings. Phase III clinical trials in platinum-sensitive patients have demonstrated its efficacy in ovarian and prostate cancers, or in combination with anti-angiogenic agents (VEGF inhibitors) in HER2-negative, BRCA1-mutant triple-negative breast cancers (TNBC), as well as in combination with Temozolomide in melanoma patients, are clear examples exhibiting adequate pharmacokinetics and clinically relevant antitumor activity. The two major obstacles to PARP inhibitor (PARPi) therapy are (i) treatment-related adverse effects, including nausea, diarrhea, alopecia, taste disturbances, fatigue, hepatotoxicity, and extensive urinary excretion, which often limit dosing and result in maximum tolerated doses (MTDs) that are insufficient to achieve optimal efficacy, and (ii) the development of therapeutic resistance, which in most cases is associated with restoring BRCA1/2 functions leading to repair of DNA, from PARPi treatment.

In this manuscript, we address two complementary aspects. First, we examine the biochemical and therapeutic implications of targeting less abundant and less well-characterized members of the PARP family, particularly mono-ADP-ribosyltransferases (MARTs) and MARylation. Second, we explore the relevance of inhibiting ADP-ribose turnover not at the level of its synthesis or covalent modification, but rather at the level of its recycling after it has fulfilled its role as a post-translational modification, focusing on poly(ADP-ribose) glycohydrolases (PARGs) and DePARylation.

Unlike DNA-dependent PARPs and tankyrases, a substantial proportion of mono-ADP-ribosyltransferases (MARTs) either lack demonstrable ADP-ribosyltransferase activity or exhibit only weak or context-dependent activity. Although these proteins have primarily been classified as mADPr-transferases, their subcellular localization and functional roles remain controversial. Nevertheless, over the past six years, an increasing body of evidence has begun to elucidate their involvement in multiple human pathologies. Notably, PARP4, PARP7, PARP10, PARP11, and PARP15 have been clearly implicated in tumor development and progression, functioning either as oncoproteins with prognostic relevance or, in certain contexts, as tumor-suppressive factors. A comprehensive study has demonstrated that inhibitors targeting MARTs (**MARTi**), including RBN-2397 (PARP7), OUL-35 (PARP10), and RBN-012759 (PARP14), exhibit favorable pharmacokinetic properties and effectively modulate key signaling pathways involved in tumor growth and metastasis, supporting their potential for future therapeutic application (Table 1). Unlike PARP inhibitors (PARPi), MART inhibitors currently exhibit low target specificity and often require high doses to achieve measurable effects. Consequently, most of these compounds lack substantial therapeutic efficacy in vivo, and their development remains largely in early experimental or preclinical stages (Table 1). On a positive note, no instances of irreducible resistance to MART inhibitors have been reported to date. Therefore, exploring synthetic lethality strategies should not hinder the clinical development of these compounds. Current research is investigating combinations with anti-angiogenics, autophagy inhibitors, or AKT/mTORC2 pathway inhibitors—key tumor hallmarks—to enhance patient sensitivity to MARTi therapy. The clinical potential of MART inhibitors is evident, but their advancement will require stronger engagement from the medical community as well as continued development of patents and novel targeted compounds.

At the terminal stage of the ADP-ribose cycle, inhibition of poly(ADP-ribose) glycohydrolases (PARGs) prevents the recycling of poly(ADP-ribose) (pADPr) modifications by blocking their endo- and exo-glycohydrolase activities. The immediate consequences include: (i) sustained ribosylation of proteins involved in transcription, DNA replication, and ADP-ribose metabolism; (ii) suppression of residual mono-ADP-ribosylation resulting from incomplete PARG-mediated processing; (iii) disruption of the ADP-ribose cycle, leading to altered NAD^+^ and cAMP recycling; and (iv) increased accumulation of DNA double-strand breaks. Collectively, these effects support the potential for the development of novel synthetic lethality strategies that may be even more effective than those based on PARP inhibition in homologous recombination–deficient contexts.

This new strategy has advanced considerably in the last five years; so much so that the development of PARG inhibitors (**PARGi**) now represents a significant achievement in terms of effectiveness. Novel chemical designs of highly selective molecules for PARG have generated numerous in vivo models and very robust data from phase I clinical trials (Table 2). Although early generations of compounds—such as intercalating agents or inhibitors that nonspecifically interfered with pADPr hydrolysis—lacked selectivity, required high concentrations to achieve reliable in vitro effects, and exhibited poor cellular permeability, the adoption of alternative strategies to target ADP-ribosylation beyond classical PARP inhibition has driven substantial progress. In recent years, this shift has enabled the development of molecules selectively targeting the catalytic site of PARG, as well as TARG and other ADP-ribose hydrolases, with improved cell permeability and minimal toxicity in both preclinical models and phase I clinical studies. Compounds such as IDE-161 and ETX-19477 highlight their potential as the future of ADP-ribosylation-targeted therapies in cancers characterized by pronounced resistance mechanisms and a high capacity to adapt to the abnormal conditions of the tumor microenvironment, including hypoxia, nutrient deprivation, immune pressure, and sustained genomic instability (Table 2).

In conclusion, this manuscript summarizes and discusses recent advances targeting two complementary processes, highlighting a novel and promising strategy to impair tumor progression.

## Figures and Tables

**Table 1:** MARTs-specific inhibitors (MARTi). Potency, Pharmacokinetics, Tumor Relevance, and Clinical Stages. Overview of the most representative MARTi, derivatives, and translational data based on clinical trial outcomes and prognostic applications.

Drug	Molecular Derivative	Target	Druggability	Tumor Relevance	Clinical Stage
ME0328 IC_50_: 0.89 µM	No	PARP3	Moderate (Selective, Limited by Doses)	High (DNA Repair, HDR)	Preclinical
RBN-2397 IC_50_: ≤3 nM	No	PARP7	High (Selective)	High (Immune Modulation, IFN-I Pathway)	Phase I
	(S)-XY-05 IC_50_: 4.5 nM	PARP7	High (Selective, Limited by Doses)	High (Immune Modulation, CD8+ Response)	Experimental
	Compound 18 IC_50_: 0.6 nM	PARP7	High (Selective, Oral Bioavailability)	High (IFN-I Pathway, Ion Channels)	Phase I
KMR-206 IC_50_: ≤10 nM	Phthtal01 IC_50_: ≤10–12 nM	PARP7	High (Non Selective, PARP1/2)	Moderate (Immune IFN-I Pathway)	No in vivo data
OUL-35 IC_50_: 329 nM	No	PARP10	Moderate (Selective)	Medium (Replication Stress)	Preclinical
OUL-3-Modified IC_50_: 130–160 nM	CycloAlkyl (8a-c)o-fluorophenyl(8h)	PARP10	Moderate (Non Selective, PARP14/15)	Moderate (Oxidative DNA Damage Stress)	Experimental
ITK7 IC_50_: 14 nM	No	PARP11	Moderate (Selective)	High (Nuclear Pore) Moderate (Replication Stress)	Preclinical
RBN012759 IC_50_: ≤3 nM	No	PARP14	High (Selective, Potent)	High (Metabolic Reprogramming, Immunotherapy)	Preclinical
RBN010860 IC_50_: ≤0.1 µM	No	PARP16	Moderate (Selective)	High (ER Stress, Mito Stress)	Preclinical

**Table 2:** PARG-specific inhibitors (PARGi). Potency, Pharmacokinetics, Tumor Relevance, and Clinical Stages. Overview of the most representative MARTi, derivatives, and translational data based on clinical trial outcomes and prognostic applications.

Drug	Molecular Activity	Druggability	Tumor Relevance and Limitations	Clinical Stage
DNA Intercalating Molecules *Proflavine*, *Ethidium Bromide*, *Ethacridine*	Covalent Bond to pADPr, Blocking hydrolysis by PARG	Low (Non-Selective)	Not Effective in Cells. High Doses; IC_50_: ≥8 µM Off-target Effects	No in vivo Data
Tannins, e.g., *Nobotanin K* IC_50_: ≥0.3 µM	ADPr analogs linking pADPr	Moderate (Non-Selective)	High Doses Low Cell Permeability	Experimental No in vivo Data
GPI-16551/GPI-18214 IC_50_: ≥2µM	Blocking the pADPr hydrolysis by PARG	Low (Toxicity)	High Doses in vitro Low Cell Permeability	Preclinical
ADP-HPD IC_50_: 0.12 µM	Chemical ADP Ribose Analog	Low (Experimental, Selective)	Inability to Penetrate Cell Membranes Restricting Activity in vitro Assays	Experimental
Rhodamine-Based PARGi (RBPIs) IC_50_: 1–6 µM	Selective Small Molecules	High (Selective)	Exacerbate DNA Damage Impermeability in Cells	Preclinical
IDE-161 https://clinicaltrials.gov/study/NCT05787587 (accessed on 10 November 2025) IC_50_: 2 nM	Selective PARG Active Site Inhibitor	High (Selective)	High (HR Deficiency, PARPi Resistance) Orally Bioavailable	Phase I/II
ETX-19477 https://clinicaltrials.gov/study/NCT06395519 (accessed on 10 November 2025) IC_50_: 0.1 µM	Selective PARG Active Site Inhibitor	High (Selective, Potent)	High (HR Deficiency, PARPi Resistance) Tolerability/Efficacy	Phase I

## Data Availability

The data and materials that support the findings of this study are available on request from the corresponding authors.

## Abbreviations

The following abbreviations are used in this manuscript:

ADPr	ADP-ribose
pADPr	Poly ADP-ribose
PARP	Poly (ADP-ribose) polymerases
MART	Mono(ADP-ribosyl) transferases
PART	Poly(ADP-ribosyl) transferases
PARG	Poly (ADP-ribose) glycohydrolases
ARHs	ADP-ribosyl) hydrolases
PARPi	PARP inhibitors/inhibition
MARTi	MART inhibitors/inhibition
PARGi	PARG inhibitors/inhibition
PAR	ADP-ribose polymer
PARylation	Poly ADP-ribosylation
MARylation	Mono ADP-ribosylation
